# Predictors of tobacco smoking among youth in an urban slum in Kampala, Uganda: A cross-sectional study

**DOI:** 10.1371/journal.pone.0321336

**Published:** 2026-02-05

**Authors:** Joyce Nakitende, Anthony Kirabira, Mona Muhammad, Elizabeth Kisembo, Denis Omara, Dennis Kalibbala, Geofrey Musinguzi, Bontle Mbongwe

**Affiliations:** 1 Department of Epidemiology and Biostatistics, School of Public Health, Makerere University, Kampala, Uganda; 2 Medical Research Council/ Uganda Virus Research Institute and London School of Hygiene and Tropical Medicine Uganda Research Unit, Entebbe, Uganda; 3 Malaria Consortium, Kampala, Uganda; 4 Infectious Diseases Institute, Kampala, Uganda; 5 School of Health Sciences, Makerere University, Kampala, Uganda; 6 Global Health Uganda, Kampala, Uganda; 7 Department of Disease Control and Environmental Health, School of Public Health, Makerere University, Kampala, Uganda; 8 Department of Environmental Health, University of Botswana, Gaborone, Botswana; All India Institute of Medical Sciences, INDIA

## Abstract

**Background:**

Tobacco use remains a significant public health concern worldwide. In Uganda, the youth use tobacco at almost three times the rate of adults, with those residing in slum areas exhibiting even higher prevalence levels. Since 2015, strict laws regulating public tobacco use have been implemented in Uganda, however, these measures have not led to a significant decline in tobacco consumption among the youths in slums.

**Objective:**

To assess the predictors of tobacco smoking among youth living in the slum areas of Kampala, Uganda.

**Methods:**

This was a cross-sectional study. It was conducted in Bwaise slum in Kampala, we recruited 422 youths aged 18–30 years. Households were sampled systematically, and quantitative data were analyzed using STATA version 14. Modified Poisson regression with robust standard errors was used, prevalence ratios (PR) were used to measure the associations. Factors were considered significant if p-values were less than 0.05.

**Results:**

The prevalence of current tobacco smoking was 52.6% while the prevalence of ever tobacco smoking was 71.6%. Most of the participants (87.4%) knew the health effects of tobacco use. Gender (adj.PR = 1.74[95% CI = 1.41–2.14]) and age (adj.PR = 1.38[95%CI = 1.10–1.74]) were the strongest predictors of tobacco smoking: the prevalence of tobacco smoking was 74% higher among males compared to females and 38% higher among those aged 21−30 years compared to their younger counterparts. Education level (adj.PR = 0.84[95%CI = 0.70–0.9]), and income/= (adj.PR = 0.79[05%CI = 0.64–0.97) were also predictive of tobacco smoking. Knowledge was also a predictor with prevalence being 34%, 29%, 42% higher among those who didn’t know that smoking causes serious illness (adj.PR = 1.34[95%CI = 1.09–1.64]), stroke (adj.PR = 1.29[95%CI = 1.06–1.59]) and lung cancer (adj.PR = 1.42[95%CI = 1.11–1.83]) respectively.

**Conclusion:**

More than half of the youth smoke tobacco despite awareness of its health effects. These findings call for development and implementation of targeted initiatives that address the unique needs and behaviors of males, aged 21–30 years, individuals of education below secondary level while addressing the knowledge gaps about effects of tobacco smoking on human health.

## Introduction

Globally, tobacco-related deaths have been estimated to exceed 8 million annually [1]. According to the World Health Organization (WHO) and the Centers for Disease Control and Prevention (CDC), this figure is projected to rise by the year 2030 [1], with Sub-Saharan Africa expected to be disproportionately affected [2]. In Uganda, approximately 10% of the population uses tobacco daily, with usage rates among the youth and young adults being significantly higher, sometimes doubling or tripling that of adults [3,4].

The most commonly used forms of tobacco include water pipe tobacco, various smokeless tobacco products, cigars, cigarillos, roll-your-own tobacco, pipe tobacco, bidis, kreteks, kuber, shisha among others [5].

Tobacco use is recognized as the leading and most significant risk factor associated with non-communicable diseases (NCDs) and other adverse health outcomes such as hypertension, oral cancers, lung cancers, chronic obstructive pulmonary disease (COPD), adverse reproductive health outcomes and sudden infant death syndrome (SIDS) [6]. Most smokers initiate their habit during adolescence and evidence suggests that as they transition into adulthood, overcoming addiction increasingly becomes difficult [7]. In Uganda, 10.5% of youth aged 13–15 used any tobacco product in 2018 [8]. Furthermore, a recent study reported a high prevalence of 36% among individuals aged 18–30 years who smoke Shisha, one of the most common and emerging tobacco products [3].

To address the global tobacco epidemic, the WHO has identified six evidence-based measures collectively referred to as “MPOWER” outlined in the Framework Convention on Tobacco Control (FCTC) [9]. They include monitoring tobacco use and prevention policies, protect people from tobacco smoke, offering assistance to quit tobacco use, warn the public about the dangers of tobacco, enforcing bans on tobacco advertising, promotion and sponsorship as well as raising taxes on tobacco.

In 2015, the Ugandan Ministry of Health (MOH) enacted a legislation that aligns with the FCTC and incorporates the essential components of the MPOWER framework. The implementation of this law is currently on ongoing [10]. However, several nationwide studies focusing on youth have reported no significant decline in tobacco use [8] and in some instances, an increase in prevalence compared to previous years [3,4].

Youth living in slums are particularly vulnerable to tobacco use largely due to the socio-economic challenges they face [11]. In Uganda, over 70% of the population consists of the youth with over 60% of the population in Kampala residing in slum conditions [12]. A slum is defined as one or a group of individuals living under the same roof in an urban area, lacking in one or more of the following five amenities”: i) durable housing; ii) sufficient living area; iii) access to improved water; iv) access to improved sanitation facilities; and v) secure tenure status and protection against forced eviction [13].The continued rise in tobacco use among this demographic, despite the presence of stringent laws warrants closer examination.

This household-based study primarily assessed the predictors of tobacco smoking among youth living in a slum in Kampala, Uganda. Understanding these dynamics is crucial for developing targeted interventions that can effectively reduce tobacco consumption in this high-risk population.

## Materials and methods

### Study design, study site and population

This was a prospective cross-sectional study in which quantitative data were collected. It was conducted within Bwaise, the largest slum settlement in Kampala, the capital city of Uganda. Kampala is divided into 5 divisions namely: Central, Makindye, Kawempe, Nakawa and Rubaga divisions. Approximately 60% of Kampala’s population resides in slum areas with a total of 57 slums distributed across these divisions [12]. Bwaise alone contains over 20,000 households and is divided into 3 large parishes each averaging around 7000 households. Bwaise is characterized by extreme poverty, high unemployment rates, elevated crime levels, inadequate sanitation and hygiene, as well as issues related to illicit drug use and prostitution, among others [14].

The study was conducted among the youth aged 18–30 who were residents of Bwaise, in Kampala city. While the United Nations (UN) defines a youth as a person between 15–24 years [15], this study adhered to the definition provided by the constitution of Uganda, which identifies youth as those aged 18–30 years [16].

### Sample size estimation

Kish Leslie’s (1965) formula for proportions [17] was used in this study to determine the required sample size. The formula is expressed as:


$$\;n=\frac{Z^{\text{2}}\;pq}{e^{\text{2}}}$$

Where n represents the required sample size, Z = 1.96 is the standard normal value corresponding to a 95% confidence level, p is the estimated prevalence of tobacco use among the youth residing in the slums of Kampala (set at 50%), q is defined as (1-p), and e is the desired level of precision (0.05). Substituting the values into the formula above; n= $\frac{{\text{1}.\text{9}\text{6}}^{\text{2}}X\text{0}.\text{5}(\text{1}-\text{0}.\text{5})}{{\text{0}.\text{0}\text{5}}^{\text{2}}}\;\;\;\;\;\;\;\text{n}\;=\;\text{384}\;\text{participants}$

Considering an anticipated non-response rate of 10%, using the expression: $\frac{Effective\;sample\;size}{\text{1}-non\;response\;rate\;anticipated}\;\;\;\;\;\;\;$ the adjusted sample size was calculated as

$\frac{\text{3}\text{8}\text{4}}{\text{1}-\text{0}.\text{1}\text{0}}\;\;\;\;\;\;\;\text{n}\;=\;\text{422}\;\text{participants}$

Thus, a total of 422 participants were targeted for the study to account for potential nonresponse.

### Sampling technique

Bwaise was selected purposively being the largest slum in Kampala district, it is divided into 3 parishes, each containing approximately 7000 households [14]. One of the parishes (parish 111) was selected randomly, to get the sampling interval, we divided 7000 (number of households in parish 111) by 422 which is 16. Therefore every 16^th^ household was selected. To get the first household, a random number between 1 and 16 was selected. From the main road, this number became the first household and subsequent households were sampled systematically until the 422-target sample size was reached.

### Inclusion and exclusion criteria

The study included youth who were: aged 18–30 years, residents of Bwaise slum, and consented to participate in the study. Participants who were deemed mentally unwell or had a known mental illness, were absent from their areas of residence at the time of data collection or were sick at the time of data collection were excluded from the study.

### Study variables

#### The dependent variable.

Current tobacco smoking served as the dependent variable in this study and was measured using standardized questions adapted from the Global Adult Tobacco Survey (GATS) and the WHO STEP-wise instrument [18,19]. According to the GATS, tobacco use is classified into categories of current tobacco users, which includes subcategories such as current smokers, daily smokers, occasional smokers and former daily or occasional smokers. Non‐tobacco users are further categorized into former daily smokers, never daily smokers and former occasional smokers. In this study, current tobacco smoking was defined as having smoked any tobacco products such as cigarettes, cigars or pipes in the past 30 days. We also secondarily assessed the lifetime prevalence of smoking (ever tobacco smoking).

#### The independent variables.

They included participants’ social demographics, and knowledge of the effects of tobacco use on health amongst the youth. Social demographics included age, gender, education level, income, and occupation among others. Knowledge of the health effects of tobacco use was assessed through questions designed to evaluate participants’ awareness and understanding of the risks associated with tobacco consumption.

### Data collection

Research assistants were trained on all study tools and ethics. Community entry was formally sought through parish 111 local leaders who granted permission to approach the households. Informed consent was sought from all eligible participants. Recruitment and data collection started on 30^th^ March 2021 and ended on 21^st^ April 2021.

### Pretesting, quality control, handling missingness

The questionnaire was pre-tested among 10 youths from *Katanga* slum and iterative revisions were made. To ensure quality control, the questionnaires were back translated from English to Luganda, the main language used in Kampala and across Bwaise. All interviews were conducted privately, particularly in a quiet and convenient place chosen by the participants. Data collected by the research assistants was checked for completeness at the end of each day. To prevent missingness of data, only trained research assistants and the principal investigator collected the data. They were all trained on how to accurately use the data collection tool and captured all the necessary data. Questionnaires were checked for any missing fields while still in the field and these were completed from there.

### Potential biases and mitigation strategies

To reduce information bias, a validated and standardized questionnaire was administered by well-trained personnel. Recall bias was mitigated by limiting the recall period to 30 days, during which participants could have used any tobacco products. To address selection bias, probabilistic systematic sampling was used to ensure that participants were representative of the general youth population. The informal nature of the setting required a degree of adaptability in applying the sampling interval, while maintaining overall methodological rigor.

### Data analysis

All data were entered into EpiData version 4.1 and subsequently exported to STATA RRID:SCR _012763 for analysis. Tobacco use characteristics and prevalence were assessed through frequency counts. The results for this objective were presented using measures of central tendency and dispersion including mean and standard deviation.

At Bivariate analysis, cross tabulations were done across all the social demographics versus current smoking and ever smoking. Both variables were stratified by gender because of its known confounding effect between tobacco smoking and other factors.

At multivariate analysis, we used modified Poisson regression with robust standard errors to evaluate significant associations between current tobacco smoking and the independent variables because the probability of our outcome of interest was above 10%. Logistics regression tends to inflate the odds ratios if the outcome is above 10%. Factors with p-values< 0.05 were considered statistically significant. We used Akaike Information Criteria (AIC) and Bayesian Information Criteria (BIC) to select the best model against other competing models. The model with the lowest AIC and BIC was selected.

### Ethical considerations

Approval to conduct the study was obtained from the Higher Degrees Research Ethics Committee at Makerere University School of Public Health. Further permission was obtained from the local community leaders. Written informed consent was obtained from every eligible participant.

### Inclusivity in global research

Additional information regarding the ethical, cultural, and scientific considerations specific to inclusivity in global research is included in the Supporting Information.

## Results

**Fig 1:**
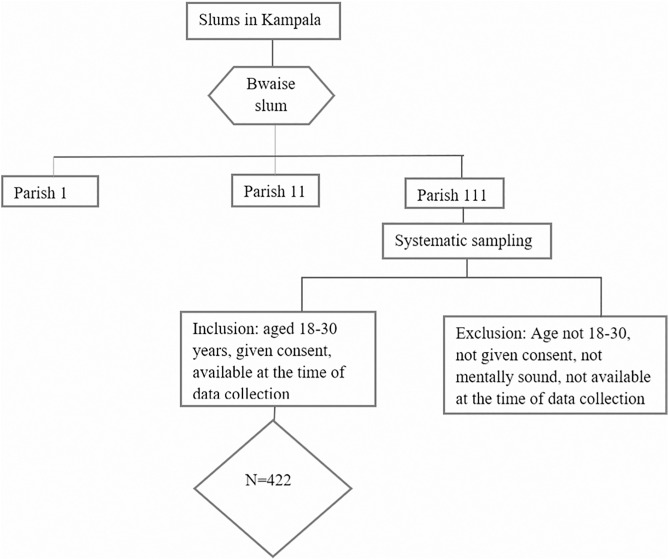
Flow diagram of participant selection and inclusion.

Approximately 7000 households were present in parish 111 from which participants were recruited. Those who were ineligible were excluded (not recorded) while those who were eligible and consented were included until the target sample size of 422 was reached, Fig 1.

### Characteristics of the study participants

A total of 422 youths from Bwaise participated in the study with 248 (58.8%) being male. The mean age of participants was 23(±3.64) with a majority (70.8%) aged 21–30 years. Approximately 80% of participants were employed. With over 42% earning less than 250,000/ = month, Table 1.

**Table 1:** Characteristics of the study participants.

	Number of respondents (n)	Proportion (%)
**Gender**		
Male	248	58.8
Female	174	41.2
**Age**		
18–20years	123	29.2
21–30years	299	70.8
**Marital status**		
Not married	314	74.4
Married	108	25.6
**Region of origin**		
Central	305	72.3
Eastern	41	9.7
Western	58	13.7
Others	18	4.3
**Education level**		
None/primary	137	32.5
Secondary/University	285	67.5
**Religion**		
Christian	226	53.6
Non-Christian	196	46.4
**Employment status**		
Employed	336	79.8
Unemployed	45	10.7
House wife	18	4.3
Students	22	5.2
**Income/month (UGX)**		
less than 250,000/=	177	41.9
250,000-500,000/=	90	21.4
500,000-750,000/=	25	5.9
750,000-1,000,000/=	14	3.3
Above 1,000,000/=	116	27.5

### Tobacco use characteristics of the study participants

Current smokers of tobacco products such as cigarettes, cigars or pipes were 222 (52.6%). Most of the youth, 302 (71.6%) had ever smoked tobacco products. The mean age at start of smoking was 17 years (± 3.91). A big proportion (57.7%) of the current smokers had tried to stop smoking in the past 12 months, Table 2.

**Table 2:** Tobacco use characteristics of the study participants.

	Frequency (n)	Percentage (%)
**Currently smoke tobacco products, such as cigarettes, cigars or pipes**		
Yes	222	**52.6**
No	200	47.4
**Currently smoke tobacco products daily (n = 222)**		
Yes	160	72.1
No	62	27.9
**Type of tobacco smoked (n = 222)**		
Manufactured cigarettes	95	42.8
Hand-rolled cigarettes	18	8.1
Pipes full of tobacco	48	21.6
Cigars, cheroots, cigarillos	4	1.8
Shisha	60	27.0
*“Kibanga”*	1145	51.8
**During the past 12 months, have you tried to stop smoking (n = 222)**		
Yes	128	57.7
No	94	42.3
**Advised to quit smoking tobacco by a health worker in the past 12 months (n = 222)**		
Yes	36	16.2
No	66	29.7
No visit during the past months	120	54.1
**In the past, did you ever smoke any tobacco products (show card)**		
Yes	80	40.0
No	120	60.0
**Currently use any smokeless tobacco such as snuff, chewing tobacco, betel**		
Yes	69	16.4
No	353	83.6
**Currently use smokeless tobacco products daily**		
Yes	44	63.8
No	25	36.2
**Ever used smokeless tobacco in the past**		
Yes	126	29.9
No	296	70.1
**Ever use smokeless tobacco daily in the past**		
Yes	53	42.1
No	73	57.9
**During the past 30 days, did someone smoke in your home?**		
Yes	194	46.0
No	228	54.0
**During the past 30 days, someone smoked in closed areas at workplace**		
Yes	322	76.3
No	62	14.7
Don’t work in a closed area	38	9.0

### Current tobacco smoking status and types of tobacco products smoked

**Fig 2:**
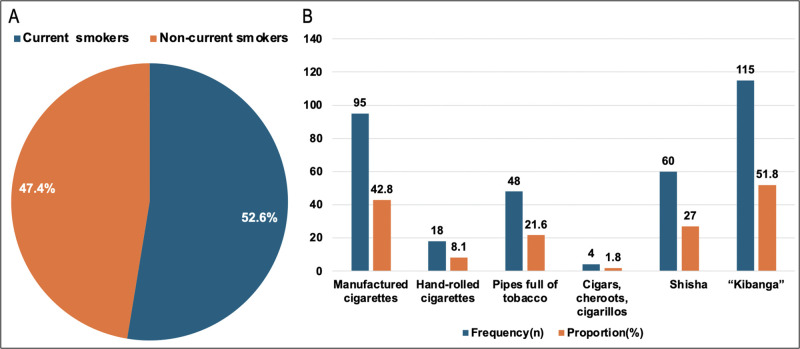
Current tobacco smoking status (A) and types of tobacco products (B) smoked by the youth.

There were 222 (52.6%) current smokers, The majority smoked *kibanga*- a local term used to mean a mixture of tobacco and marijuana. Only 4 (1.8%) participants smoked cigar, cheroots or cigarillos. Most participants used more than one tobacco product, therefore the (n) don’t add up to (n = 222), **Fig 2**.

### Knowledge of the health effects of tobacco smoking

Overall, most participants knew the effects of tobacco on health. More than 3/4 knew that tobacco causes serious illnesses, lung cancer and that smokeless tobacco causes serious illnesses. While less than 1/2 knew that smoking tobacco causes stroke (Table 3).

**Table 3:** Knowledge of the health effects of tobacco smoking.

	Number of respondents (n)	Proportion (%)
**Knows or believes that smoking tobacco causes serious illness**		
Yes	369	87.4
No	30	7.1
Don’t know	23	5.5
**Knows or believes that smoking tobacco causes Stroke (blood clots in the brain that may cause paralysis)**		
Yes	189	44.8
No	85	20.1
Don’t know	148	35.1
**Knows or believes that smoking tobacco causes Heart attack**		
Yes	278	65.9
No	64	15.1
Don’t know	80	19.0
**Knows or believes that smoking tobacco cause Lung cancer**		
Yes	383	90.8
No	14	3.3
Don’t know	25	5.9
Knows or believes that using *smokeless tobacco* causes serious illness		
Yes	305	72.3
No	41	9.7
Don’t know	76	18.0

### Cross tabulation of social demographics across smoking status

On cross tabulating current smokers versus each demographic gender (p < 0.001), age (p = 0.002). Region of origin, education level, employment status and income were statistically significant. On stratification, age was not significant. Among those who had ever smoked, region of origin (p < 0.001), religion, employment status (p < 0.001) and income were statistically significant (Table 4).

**Table 4:** Cross tabulation results of social demographics across smoking status.

	Current smoker			Current smoker by gender			Ever smoked		
	Yes	No	P-value	Males	Females	P-value	Males	Females	P-value
	N = 222	N = 200		(n = 158)	(n = 64)		(n = 208)	(n = 94)	
	n(%)	n(%)		n (%)	n (%)		n (%)	n (%)	
**Gender**			0.001*						
Males	158(71.17)	90(45.00)							
Females	64(28.83)	110(55.0)							
**Age**			0.002*			0.616			0.616
18-20years	73(36.50)	50(22.52)		37(23.4)	13(20.3)		54(26.0)	27(28.7)	
21-30years	127(63.50)	172(77.48)		121(76.6)	32(79.7)		154(74.0)	67(71.3)	
**Marital status**			0.883			0.847			0.78
Not married	164(73.87)	149(74.50)		118(74.7)	47(73.4)		158(76.0)	70(74.5)	
Married	58(26.13)	51(25.50)		40(25.3)	17(26.6)		50(24.0)	24(25.5)	
**Region of origin**			0.012*			0.010*			<0.001*
Central Uganda	172(77.48)	133(66.50)		131(82.9)	41(64.1)		174(83.7)	57(60.6)	
Outside central	50(22.52)	67(33.50)		27(17.1)	18(35.9)		34(16.3)	37(39.4)	
**Education level**			0.004*			0.059			0.192
None/primary	86(38.74)	51(25.63)		55(34.8)	31(48.4)		68(32.7)	38(40.4)	
Secondary/Univ	136(61.26)	148(74.37)		103(65.2)	33(51.6)		140(67.3)	56(59.6)	
**Religion**			**0.241**			0.004*			**0.002**
Christian	115(51.80)	115(57.50)		70(44.3)	42(65.6)		94(45.2)	61(64.9)	
Non-Christian	107(48.20)	85(42.50)		88(55.7)	22(34.4)		114(54.8)	33(35.1)	
**Employment status**			0.003*			<0.001*			<0.001*
Employed	195(87.84)	154(77.00)		144(91.7)	48(75.0)		188(90.8)	69(73.4)	
Unemployed	27(12.16)	46(23.00)		13(8.3)	16(25.0)		19(9.2)	25(26.6)	
**Income (UGX)**			0.037*			0.008*			0.004*
>250,000	115(51.80)	73(36.50)		70(44.3)	39(60.9)		85(40.9)	49(52.2)	
250,000-500,000	53(23.87)	53(26.50)		41(25.9)	6(9.4)		55(26.4)	11(11.7)	
500,000-750,000	12(5.41)	17(8.50)		10(6.3)	1(1.6)		16(7.7)	3(3.1)	
750,000–1 M	9(4.05)	10(5.00)		5(3.2)	0(0.0)		8(3.9)	1(1.1)	
Above 1,000,000	11(4.95)	18(9.00)		32(20.3)	18(28.1)		44(21.1)	30(31.9)	

*Statistically significant at 5% level of significance, *p < 0.1, **p < 0.05, ***p < 0.001, Chi square tests were used as the measure of association. The ever smoked category (n = 302) includes both past smokers (n = 80) and current tobacco smokers(n = 222)

### Cross-tabulation of knowledge by smoking status

Across current smokers and non-smokers, knowing that smoking causes serious illness, stroke, lung cancer, heart attacks were all significant, on stratifying current smokers by gender, only knowledge that smokeless tobacco causes serious illness was statistically significant (Table 5).

**Table 5:** Knowledge of Tobacco Effects by Smoking Status.

	Smoking status			Current smoker by gender			Ever Smoked		
	Current smoker	Non-current smoker	P-value	Males	Females	P-value	Males	Females	p-value
**(N = 222)**	**(N = 200)**	**(n =** **158)**	**(n =** **64)**	**(n = 208)**	**(n = 94)**
**n (%)**	**n (%)**	**n(%)**	**n(%)**	**n (%)**	**n (%)**
**Knows that smoking causes serious illness**			P < 0.000*			0.112			0.105
Yes	179(80.63)	190(95.00)		126(79.7)	53(82.8)		174(83.7)	82(87.2)	
No	27(12.16)	3(1.50)		23(14.6)	4(6.3)		23(11.1)	4(4.3)	
Don’t know	16(7.21)	7(3.50)		9(5.7)	7(10.9)		11(5.3)	8(8.5)	
**Knows that smoking causes Stroke**			P < 0.000*			0.642			0.563
Yes	88(39.64)	101(50.50)		64(40.5)	24(37.5)		89(42.8)	41(43.6)	
No	61(27.48)	24(12.00)		45(28.5)	16(25.0)		53(25.5)	19(20.2)	
Don’t know	73(32.88)	75(37.50)		49(31.0)	24(37.5)		66(31.7)	34(36.2)	
**Knows that smoking causes Heart attack**			P < 0.000*			0.791			0.548
Yes	132(59.46)	146 (73.00)		93(58.9)	39(60.9)		134(64.4)	64(68.1)	
No	48(21.62)	16(8.00)		36(22.8)	12(18.8)		37(17.8)	12(12.8)	
Don’t know	42(18.92)	38 (19.00)		29(18.3)	13(20.3)		37(17.8)	18(19.1)	
**Knows that smoking cause Lung cancer**			P < 0.000*			0.501			0.276
Yes	189(85.14)	193(96.50)		137(86.7)	53(82.8)		186(89.4)	81(86.2)	
No	12(5.41)	2(1.00)		9(5.7)	3(4.7)		10(4.8)	3(3.2)	
Don’t know	21(9.46)	5(2.50)		12(7.6)	8(12.5)		12(5.8)	10(10.6)	
**Knows that SLT causes serious illness**			0.008*			0.017*			0.095
Yes	147(66.22)	158(79.00)		112(70.9)	35(54.7)		147(70.7)	58(61.7)	
No	29(13.06)	12(6.00)		21(13.3)	8(12.5)		26(12.5)	10(10.6)	
Don’t know	46 (20.72)	30(15.00)		25(15.8)	21(32.8)		35(16.8)	26(27.7)	

*Statistically significant at 5% level of significance, *p < 0.1, **p < 0.05, ***p < 0.001, Chi square tests were used as the measure of association. The ever-smoked category (n = 302) includes both past smokers (n = 80) and current tobacco smokers(n = 222)

### Predictors of tobacco smoking

After adjusting for other factors, the prevalence of tobacco smoking was 74% higher among males compared to females (adj.PR = **1.74[95% CI = 1.41–2.14])**, 38% higher among those aged 21–30 years compared to their younger counterparts (adj.PR = **1.38[95%CI = 1.10–1.74])**, 26% lower among those with secondary/university level education, 21% lower among those who earned between 250,000–500,000/ = , 34%, 29%, 42% higher among those who didn’t know that smoking causes serious illness, stroke and lung cancer respectively. On stratification by gender: females between 21–30 years had 83% higher prevalence of smoking (adj.PR = **1.83[95%CI = 1.14–2.94])** compared to males who only had 34% higher prevalence than their younger counterparts. Females with secondary/university level education had 40% lower prevalence of smoking compared to their counterparts with primary or no education and females who didn’t know that smokeless tobacco causes serious illness had 37% higher prevalence of smoking compared to their counterparts (adj PR = **2.37[95% CI = 1.33–4.23])** (Table 6).

**Table 6:** Multivariate analysis results showing predictors of tobacco smoking.

Variable	Overall multivariate analysis		Stratified multivariate analysis	
	All (N = 422)	All (N = 422)	Male (N=)	Female (N=)
	uPR(95% CI)	aPR(95% CI)	aPR(95% CI)	aPR(95% CI)
**Gender**				
Female	**1**	**1**		
Male	1.73(1.39-2.15)***	1.74(1.41-2.14)***	**–**	–
**Age**				
18–20years	**1**	**1**	**1**	**1**
21–30years	1.42(1.12-1.79)**	1.38(1.10-1.74)**	1.34(1.06-1.70)*	1.83(1.14-2.94)*
**Marital status**				
Not married	1	------	----	1
Married	1.02(0.83-1.25)	------	----	-----
**Region of origin**				
Central	**1**	------	-----	----
Outside Central	0.76(0.60-0.96)*	------	-----	**----**
**Education level**				
None/primary	**1**	**1**		1
Secondary/University	0.76(0.64-0.91)**	0.84(0.70-0.99)*	-----	0.60(0.42-0.86)**
**Religion**				
Christian	1	------	----	---
Non-Christian	1.11 (0.93-1.33)	------	----	---
**Employment status**				
Employed	**1**	------	----	---
Unemployed	0.66(0.48-0.91)*	------	----	---
**Income (/=)**				
Less than 250,000/=	**1**	1	----	---
250,000-500,000/=	0.82(0.65-1.02)	0.79(0.64-0.97) *	----	---
500,000-750,000/=	0.68(0.43-1.06)	0.68(0.45-1.04)	----	---
750,000-1,000,000/=	0.77(0.48-1.26)	0.81(0.52-1.27)	----	---
Above 1,000,000/=	0.62(0.38-1.00)*	0.66(0.41-1.06)	----	-----
**Knows smoking causes:**				
**Serious illness**				
Yes	1	1	**1**	
No	1.85(1.58-2.16)***	1.34(1.09-1.64)**	1.48 (1.22-1.79)***	**----**
I don’t Know	1.4(1.07-1.92)*	1.12(0.81-1.55)	1.08 (0.74-1.57)	**----**
**Stroke**				
Yes	1	1		
No	1.54(1.26-1.89)***	1.29(1.06-1.59)*	----	
I don’t know	1.06(0.39-0.54)	1.02(0.824- 1.25)	----	
**Heart attack**				
Yes	1			
No	1.56(1.31- 1.91)	-----	----	1.83(1.159-2.89)**
I don’t know	1.11(0.89-1.41)	-----	----	1.236(0.79-1.93)
**Lung cancer**				
Yes	1			
No	1.73(1.37- 2.19)***	1.03(0.76-1.41)	0.98(0.720-1.35)	-----
I don’t know	1.63(1.32-2.02)***	1.42(1.11-1.83)**	1.35(1.07-1.69)*	-----
**SLT causes illness**				
Yes	1			
No	1.467(1.17-1.85)**	**-----**	**------**	2.37(1.33- 4.23)**
I don’t know	1.26(1.01-1.56)*	**-----**	**------**	2.04(1.38- 3.00)***

uPR = Unadjusted Prevalence Ratio, aPR = adjusted Prevalence Ratio, CI = Confidence Interval *Statistically significant at 5% level of significance, *p < 0.1, **p < 0.05, ***p < 0.001. PR = Prevalence Ratio, CI = Confidence Interval

## Discussion

We found that more than 5 in 10 youths who dwell in this slum smoked tobacco. Approximately similar results were obtained in Uganda in a survey of patients living with HIV [20], among males who work in a police barracks [21], and among pregnant women in Maracha district [22]. Similarly in Palestine, the reported prevalence of tobacco use was 47.7% [23], and 42.3% in a study among young men in Bangladesh [24]. On the contrary, this is slightly higher than the prevalence reported by the recent estimates for Uganda in the GYTS survey [4]. Similarly, the GATS estimates for Uganda reported 1 in 10 prevalence of smoking in the general population [3,25]. This can be attributed to many factors: For the estimates from GYTS (Uganda), the definition of youths was different from that used in our study (Ugandan constitution definition of a youth 18–30 years were included in our study). For the estimates from the GATS, that survey was not specific to slum areas but rather to the general Ugandan population. The high level of prevalence in our study can thus be attributed to the nature of the study site, a slum setting characterized by high crime rate, poverty in extremes, and rampant use of substances of abuse.

Unlike in most studies done worldwide [23,26], we found that the most common form of tobacco used was termed as “*Kibanga*”, a local term used to mean a mixture of tobacco and marijuana, hand rolled to form a stick of mixed substances. Literature about the use of ‘*kibanga’* is limited, however from qualitative interviews, of unpublished work, it was mentioned that this mixture of marijuana and tobacco was safe, neutral and had no effects on human health whatsoever. Elsewhere, it has been documented to help with relaxation and bad moods [27].

We also found out that several participants who consumed tobacco at most times narrated to have firstly had chewed mira- plant leaves abused in Uganda. Similar findings were documented in other studies [28–30].

We found that gender was a strong predictor of tobacco smoking, with the prevalence of smoking being 74% higher among males compared to females. Evidence globally suggests that males have higher smoking levels compared to women, mostly linked to peer pressure and social reasons [31–33]. Similar evidence was documented in the recent GATS survey in Uganda [25]. On the other hand, there is very scanty literature suggesting otherwise.

Tobacco smoking increased with increase in age, older participants [21–30] used tobacco much more than their younger counterparts. Similar findings were reported in other studies [3,34,35]. Studies report that most individuals who start smoking rarely stop the habit. The reason for this remains complex, but it has been linked to addiction to nicotine, increasing stress as one grows and peer pressure

Education level was also a predictor of tobacco smoking, participants who attained secondary or university level education had 26% lower prevalence of smoking, furthermore, females had 40% lower prevalence of smoking compared to males. This is consistent with findings from elsewhere [35,36]. Education has been recognized as a key determinant of socio-economic status [37] with tobacco use among individuals with lower education levels often reflecting conditions shaped by stress, financial hardship, and poverty -factors that collectively increase tobacco use.

We found knowledge to be a strong predictor of tobacco smoking among the youth in this study. Participants who didn’t know that smoking causes serious illness, stroke, lung cancer and that smokeless tobacco causes serious illness all had significantly higher prevalence of smoking compared to their counterparts who knew or believed that smoking has ill health effects towards one’s health. This is consistent with other studies elsewhere [38,39]. This behavior mirrors a psychological function associated with a diminished perception of threat. But generally, most of the participants in this study had good knowledge about the ill effects of tobacco, much as they were smokers. These findings are consistent with those from Bangladesh [40] and New Delhi [41]. Persistent tobacco smoking even when one is aware of its ill effects may be explained by several cessation barriers such as lack of social support and continued peer pressure. The WHO advises that there are still more opportunities to cover smoking knowledge gaps [42].

### Limitations

This was a cross-sectional study, it couldn’t infer causality. Tobacco use was self-reported; it may thus be underestimated due to social desirability bias. While systematic sampling was intended and largely maintained, practical challenges in the informal nature of the settings could have led to some irregularities in the sampling process, which may have influenced the consistency of sample selection.

## Conclusion

More than half of the youth smoked tobacco despite the majority knowing the health effects of tobacco use. Future smoking control interventions for most at risk populations should target males, aged 21–30 years, of primary or no education, and as well address knowledge gaps regarding tobacco effects on health.

## Supporting information

S1 File Inclusivity in global health research checklist. (DOCX)

S2 File Data set. (CSV)

## References

[1] World Health Organization. WHO report on the global tobacco epidemic, 2023: protect people from tobacco smoke. 2023.

[2] Méndez D, Alshanqeety O, Warner KE. The potential impact of smoking control policies on future global smoking trends. Tob Control. 2013;22(1):46–51. doi: 10.1136/tobaccocontrol-2011-050147 22535364

[3] Aanyu C, Kadobera D, Apolot RR, Kisakye AN, Nsubuga P, Bazeyo W, et al. Prevalence, knowledge and practices of shisha smoking among youth in Kampala City, Uganda. Pan Afr Med J. 2019;32:61. doi: 10.11604/pamj.2019.32.61.15184 31223353 PMC6560999

[4] Kadobera D, Chaussard M, Lee KA, Ayebazibwe N, Ndyanabangi S. Changes in prevalence of tobacco use and the factors that may affect use among Uganda youth: the Global Youth Tobacco Survey (GYTS) 2007-2011. Pan Afr Med J. 2016;25:152. doi: 10.11604/pamj.2016.25.152.9991 28292114 PMC5326025

[5] World Health Organization. Tobacco fact sheet: World Health Organisation; 2023 https://www.who.int/news-room/fact-sheets/detail/tobacco

[6] World Health Organisation. The Tobacco Body. 2019. https://iris.who.int/bitstream/handle/10665/324846/WHO-NMH-PND-19.1-eng.pdf

[7] World Health Organisation. Adolescent and young adult health [Report]. 2024 https://www.who.int/news-room/fact-sheets/detail/adolescents-health-risks-and-solutions

[8] Ministry of Health Uganda. Global Youth Tobacco Survey,Uganda,2018. 2018. https://extranet.who.int/ncdsmicrodata/index.php/catalog/812

[9] World Health Organisation. WHO report on the global epidemic 2023: Protect people from Tobacco smoke. World Health Organisation; 2023. https://iris.who.int/bitstream/handle/10665/372043/9789240077164-eng.pdf?sequence=1

[10] Government of Uganda. Uganda Tobacco control Act. 2015. https://bills.parliament.ug/attachments/Laws%20of%20Uganda%20(Acts)%20-%20THE%20TOBACCO%20CONTROL%20ACT,%202015.pdf

[11] Renzaho AMN, Kamara JK, Kamanga G. The Ugandan Youth Quality of Life index: Assessing the relevance of incorporating perceived importance into the quality of life measure and factors associated with the quality of life among youth in slum areas of Kampala, Uganda. Glob Health Action. 2016;9:31362. doi: 10.3402/gha.v9.31362 27427302 PMC4947833

[12] Uganda Bureau of Statistics. Uganda National Household Survey 2019/2020. Kampala, Uganda: Uganda Bureau of Statistics. 2021.

[13] UN-Habitat U. State of the world’s cities 2006/7. US: Earthscan. 2006.

[14] Uganda Go. kampala city slum profiling-Kawempe municipality. 2014.

[15] United Nations. Definition of the youth 2013. https://www.un.org/esa/socdev/documents/youth/fact-sheets/youth-definition.pdf

[16] Government of Uganda. National Youth Council Act, chapter 319. 2010. http://www.parliament.go.ug

[17] Kish L. Survey sampling. 1965.

[18] World Health Organisation. Global Adult Tobacco Survey 2025. https://www.who.int/teams/noncommunicable-diseases/surveillance/systems-tools/global-adult-tobacco-survey

[19] World Health Organisation. STEPwise approach to NCD risk factor surveillance (STEPS). 2025.

[20] Mdege ND, Makumbi FE, Ssenyonga R, Thirlway F, Matovu JKB, Ratschen E, et al. Tobacco smoking and associated factors among people living with HIV in Uganda. Nicotine Tob Res. 2021;23(7):1208–16. doi: 10.1093/ntr/ntaa262 33295985 PMC7610955

[21] Basaza R, Otieno E, Musinguzi A, Mugyenyi P, Haddock CK. Factors influencing cigarette smoking among soldiers and costs of soldier smoking in the work place at Kakiri Barracks, Uganda. Tob Control. 2017;26(3):330–3. doi: 10.1136/tobaccocontrol-2015-052878 27165996 PMC5104672

[22] Alege JB, Jurua RO, Drazidio J. Prevalence of tobacco use and associated risk factors among pregnant women in Maracha District, Uganda. 2021. 10.5897/jphe2020.1276 PMC9938523 36819909

[23] Abu Seir R, Kharroubi A, Ghannam I. Prevalence of tobacco use among young adults in Palestine. 2020. 10.26719/2020.26.1.75 32043549

[24] Kelishadi R, Mokhtari MR, Tavasoli AA, Khosravi A, Ahangar-Nazari I, Sabet B, et al. Determinants of tobacco use among youths in Isfahan, Iran. Int J Public Health. 2007;52(3):173–9. doi: 10.1007/s00038-007-6017-x 17958284

[25] Ministry of Health(MOH) U. Global Adult Tobacco Survey,Uganda. Uganda; 2013. https://health.go.ug/sites/default/files/GATS%20Uganda%20Fact%20Sheet%2C%20June%2012%202014.pdf

[26] Ministry of Health. Global Adult Tobacco Survey 2013 fact sheet; Uganda. 2014 .https://extranet.who.int/ncdsmicrodata/index.php/catalog/868/related-materials

[27] Pedersen ER, Tucker JS, Davis JP, Dunbar MS, Seelam R, Rodriguez A, et al. Tobacco/nicotine and marijuana co-use motives in young adults: Associations with substance use behaviors one year later. Psychol Addict Behav. 2021;35(2):133–47. doi: 10.1037/adb0000638 32551726 PMC7746603

[28] Kassim S, Rogers N, Leach K. The likelihood of khat chewing serving as a neglected and reverse “gateway” to tobacco use among UK adult male khat chewers: A cross sectional study. BMC Public Health. 2014;14:448. doi: 10.1186/1471-2458-14-448 24885131 PMC4039549

[29] Guliani H, Gamtessa S, Çule M. Factors affecting tobacco smoking in Ethiopia: Evidence from the demographic and health surveys. BMC Public Health. 2019;19(1):938. doi: 10.1186/s12889-019-7200-8 31299938 PMC6624889

[30] Deressa Guracho Y, Addis GS, Tafere SM, Hurisa K, Bifftu BB, Goedert MH, et al. Prevalence and factors associated with current cigarette smoking among ethiopian university students: A systematic review and meta-analysis. J Addict. 2020;2020:9483164. doi: 10.1155/2020/9483164 32373383 PMC7191364

[31] Waldron I, Bratelli G, Carriker L, Sung WC, Vogeli C, Waldman E. Gender differences in tobacco use in Africa, Asia, the Pacific, and Latin America. Soc Sci Med. 1988;27(11):1269–75. doi: 10.1016/0277-9536(88)90357-7 3206258

[32] Global Youth Tobacco Survey Collaborating Group. Differences in worldwide tobacco use by gender: findings from the Global Youth Tobacco Survey. J Sch Health. 2003;73(6):207–15. doi: 10.1111/j.1746-1561.2003.tb06562.x 12899101

[33] Al-Hamdani M, Hopkins DB, Hardardottir A, Davidson M. Perceptions and Experiences of Vaping Among Youth and Young Adult E-Cigarette Users: Considering Age, Gender, and Tobacco Use. J Adolesc Health. 2021;68(4):787–93. doi: 10.1016/j.jadohealth.2020.08.004 32943292

[34] Ngaruiya C, Abubakar H, Kiptui D, Kendagor A, Ntakuka MW, Nyakundi P, et al. Tobacco use and its determinants in the 2015 Kenya WHO STEPS survey. BMC Public Health. 2018;18(Suppl 3):1223. doi: 10.1186/s12889-018-6058-5 30400915 PMC6219013

[35] Mbongwe B, Tapera R, Phaladze N, Lord A, Zetola NM. Predictors of smoking among primary and secondary school students in Botswana. PLoS One. 2017;12(4):e0175640. doi: 10.1371/journal.pone.0175640 28414757 PMC5393585

[36] Ngaruiya C, Abubakar H, Kiptui D, Kendagor A, Ntakuka MW, Nyakundi P, et al. Tobacco use and its determinants in the 2015 Kenya WHO STEPS survey. BMC Public Health. 2018;18(Suppl 3):1223. doi: 10.1186/s12889-018-6058-5 30400915 PMC6219013

[37] Trias-Llimós S, Spijker JJ, Blanes A, Permanyer I. Age and cause-of-death contributions to educational inequalities in life expectancy and lifespan variation in a low-mortality country: a cross-sectional study of 1.67 million deaths in Spain (2016–19). SSM-Population Health. 2023;23:101461. 37554668 10.1016/j.ssmph.2023.101461 PMC10404554

[38] Nurmansyah MI, Umniyatun Y, Jannah M, Syiroj AT, Hidayat DN. Knowledge, attitude and practice of cigarette smoking among senior secondary school students in Depok, Indonesia. Int J Adolesc Med Health. 2019;33(2):10.1515/ijamh-2018–0124. doi: 10.1515/ijamh-2018-0124 30913035

[39] Swain P, Singh P. Assessment of knowledge and practice towards tobacco and alcohol consumption among male adolescents in urban slums of Delhi. JPMHH. 2020;6(1):10–5. doi: 10.18231/j.jpmhh.2020.003

[40] Abbet T. Covid-19 vaccines will expire in July, says health ministry. The Daily Monitor. 2021.

[41] Mohanan P, Swain S, Sanah N, Sharma V, Ghosh D. A study on the prevalence of alcohol consumption, tobacco use and sexual behaviour among adolescents in urban areas of the Udupi District, Karnataka, India. Sultan Qaboos Univ Med J. 2014;14(1):e104-12. doi: 10.12816/0003343 24516739 PMC3916261

[42] Smith H, Ameh C, Roos N, Mathai M, Broek N van den. Implementing maternal death surveillance and response: A review of lessons from country case studies. BMC Pregnancy Childbirth. 2017;17(1):233. doi: 10.1186/s12884-017-1405-6 28716124 PMC5513145

